# Presence of Virus Neutralizing Antibodies in Cerebral Spinal Fluid Correlates with Non-Lethal Rabies in Dogs

**DOI:** 10.1371/journal.pntd.0002375

**Published:** 2013-09-19

**Authors:** Clement W. Gnanadurai, Ming Zhou, Wenqi He, Christina M. Leyson, Chien-tsun Huang, Gregory Salyards, Stephen B. Harvey, Zhenhai Chen, Biao He, Yang Yang, D. C. Hooper, Berhnard Dietzchold, Zhen F. Fu

**Affiliations:** 1 Department of Pathology, College of Veterinary Medicine, University of Georgia, Athens, Georgia, United States of America; 2 Department of Population Health, College of Veterinary Medicine, University of Georgia, Athens, Georgia, United States of America; 3 Department of Infectious Diseases, College of Veterinary Medicine, University of Georgia, Athens, Georgia, United States of America; 4 State-key Laboratory of Agricultural Microbiology, College of Veterinary Medicine, Huazhong Agricultural University, Wuhan, China; 5 Departments of Cancer Biology and Neurological Surgery, Thomas Jefferson University, Philadelphia, Pennsylvania, United States of America; Swiss Tropical and Public Health Institute, Switzerland

## Abstract

**Background:**

Rabies is traditionally considered a uniformly fatal disease after onset of clinical manifestations. However, increasing evidence indicates that non-lethal infection as well as recovery from flaccid paralysis and encephalitis occurs in laboratory animals as well as humans.

**Methodology/Principal Findings:**

Non-lethal rabies infection in dogs experimentally infected with wild type dog rabies virus (RABV, wt DRV-Mexico) correlates with the presence of high level of virus neutralizing antibodies (VNA) in the cerebral spinal fluid (CSF) and mild immune cell accumulation in the central nervous system (CNS). By contrast, dogs that succumbed to rabies showed only little or no VNA in the serum or in the CSF and severe inflammation in the CNS. Dogs vaccinated with a rabies vaccine showed no clinical signs of rabies and survived challenge with a lethal dose of wild-type DRV. VNA was detected in the serum, but not in the CSF of immunized dogs. Thus the presence of VNA is critical for inhibiting virus spread within the CNS and eventually clearing the virus from the CNS.

**Conclusions/Significance:**

Non-lethal infection with wt RABV correlates with the presence of VNA in the CNS. Therefore production of VNA within the CNS or invasion of VNA from the periphery into the CNS via compromised blood-brain barrier is important for clearing the virus infection from CNS, thereby preventing an otherwise lethal rabies virus infection.

## Author Summary

Inexorable lethality is still commonly attributed to rabies infection, although there is increasing evidence for non-lethal infection and even recovery from clinical rabies in various animal species and humans. This paper reports non-lethal infection in dogs. The striking difference between dogs that survived a wt RABV infection and dogs that succumbed to the infection is that the surviving dogs showed high level of VNA in the serum and in the CSF, as well as mild immune cell accumulation in the CNS, whereas dogs that succumbed to disease showed little or no VNA in the serum or in the CSF and developed severe CNS inflammation. Considering the role of VNA in clearing the virus from the CNS, production of VNA within the CNS or infiltration of VNA from the periphery into the CNS across the blood-brain barrier appears to be important for clearing the virus from CNS thereby preventing a lethal rabies infection.

## Introduction

Rabies is a highly lethal disease caused by the neurotropic rabies virus (RABV). It has been estimated that about 55,000 persons died from rabies each year mostly in Africa and Asia [1]. Successful vaccines have been developed for the prophylaxis of the disease. Timely post-exposure prophylaxis (PEP) can prevent the development of rabies, when individuals are exposed to the virus. Unfortunately, PEP is ineffective once clinical signs have appeared and virus replicates in the CNS [2], [3]. It is generally believed that virus clearance is impossible once the virus reaches the brain [4], [5]. However, there is now increasing evidence that non-lethal infection can occur in various animal species and in humans [6]–[8]. Up to date, six non-lethal human rabies cases have been documented in the US alone [9]–[13]. All these patients either had rabies specific antibodies in the cerebral spinal fluids (CSF) at the time of hospitalization or after treatment with the Milwaukee Protocol or a modification thereof [14]. In addition, recovery of laboratory animals from clinical rabies has also been reported [15]. It has been demonstrated in mice that the clearance of the virus from the CNS requires the induction of innate immune responses in the CNS, induction of RABV-specific adaptive immunity in the periphery, as well as infiltration of immune effectors across the blood-brain barriers (BBB) [16]. Roy et al. have demonstrated that the lethality of infection with the silver-haired bat RABV can be reduced by opening the BBB. Failure to enhance BBB permeability prevents the delivery of immune effectors to the CNS leading to the lethal outcome of rabies infection [17]–[19]. Although chronic natural RABV infection in vampire bats [20], recovery from experimental RABV infection in dogs and ferrets, and recovery of humans from rabies has been documented [10], [13], [21], the mechanism(s) involved in the prevention of lethal rabies are not completely understood. These observations have led to renewed efforts to obtain evidence of underlying mechanisms behind nonfatal rabies infections. One of the major findings is that non-lethal wt RABV infection or recovery from rabies correlates with the presence of VNA in the CSF that presumably crossed the BBB [8], [19], [22].

In the present report, we describe the observation of non-lethal infection in dogs after experimental infection with a wild type (WT) RABV that originated from a dog (DRV-Mexico) [23]. We found that the non-lethal infection correlated with the presence of high level VNA in the CSF, in contrast to lethal infection, where no or only little VNA (<0.5 IU) were detected in the CSF. On the other hand, vaccinated dogs resisted a challenge infection with no detectable VNA in the CSF but high VNA levels in the serum.

## Methods

### Viruses, animals, and reagents

Dog RABV (DRV-Mexico) was originally isolated from a dog of Mexico origin [23], [24]. Virus stocks were prepared by inoculating 10 µl of the virus by the intracerebral route into one-day-old suckling mice. When moribund, the mice were euthanized and brains removed. A 10% (w/v) suspension was prepared by homogenizing the brain in DMEM. The homogenate was centrifuged to remove debris and the supernatant collected and stored at −80°C. Healthy, non-rabies vaccinated, 5 month old clinically healthy female beagles were obtained from Covance, USA. All the experimental dogs were housed individually in temperature- and light-controlled quarters in the Animal Facility, College of Veterinary Medicine at University of Georgia. All animal experiments were carried out under Institutional Animal Care and Use Committee-approved protocols (animal welfare assurance number A3085-01).

### Dog vaccination and infection

All the experimental dogs were pre-screened for the presence of maternal VNA to RABV using the Rapid Fluorescent Focus Inhibition Test (RFFIT). Dogs were sedated with Acepromazine, an IM injection of phenothiazine derivative. Eight dogs were randomly selected and infected intramuscularly (i.m) with 100 µl viral suspension containing 200 MICLD_50_ (50% mouse intracerebral lethal dose) of DRV-Mexico by direct inoculation into the left hemisphere of the temporalis muscle. Another group of 4 dogs were immunized with a RABV vaccine. The immunized dogs were challenged after 4 weeks post immunization with 100 µl of DRV suspension as described above. Dogs were observed at least once a day prior to challenge and two to four times a day for 30 days after challenge. Humane endpoint of the study is the appearance of hind limb paralysis of one or both limbs and the experimental endpoint of the study was decided on the basis of observed clinical signs for 30 days post challenge. Blood, CSF and brain samples were collected before infection and/or at the time of termination for various analyses including complete blood counts (CBC), serum biochemistry, histopathology, immunohistochemistry, antibody titration, and CSF cytology.

### Histopathology and immunohistochemistry

For histopathology and immunohistochemistry, brain samples were fixed in 10% neutral buffered formalin as described previously [25]. Brains were removed and paraffin embedded for coronal sections. For de-paraffinization, slides were heated at 60°C for 25 min and then dipped in CitriSolv (Fisher Scientific, PA) three times for 5 min and dried until chalky white. The slides were then stained with hematoxylin and eosin (H&E). Slides were heated in antigen unmasking solution (Vector Laboratories, CA) above 90°C for 20 min and allowed to cool down to room temperature. Anti-RABV N monoclonal antibody Mab N42 and Anti-RABV G Mab 53 were used to detect the viral antigens N and G, respectively [26]. Biotinylated secondary antibodies were used for detection as described [27], with avidin–biotin–peroxidase complex (Vector Laboratories, CA) and diaminobenzidine (DAB) as a substrate for color development. The intensity of DAB signals corresponding to CD3 antigen was quantified manually to obtain statistical analysis. Both the histopathology and immunohistochemistry slides were read and interpreted by the same pathologist.

### Clinical pathology

The blood and CSF samples were collected before and after infection and sent to the Clinical Pathology Laboratory at University of Georgia for analysis. Blood samples were collected in plastic tubes coated with or without EDTA anticoagulant from the jugular vein or cephalic veins. Whole blood and serum were analyzed for hematology parameters and chemistry parameters, respectively. CBCs were performed on Siemens Bayer Advia 120 using flow cytometry laser light scatter methodology. Serum chemistry is performed on Roche Hitachi P Module analyzer. The CSF samples were collected from the cerebellomedullary cistern site and were evaluated for various inflammatory parameters, such as, white blood cell counts (WBC) and total protein concentration. CSF cytology is performed by counting the cells on a Neubauer Hemocytometer and a cytospin smear is made to differentiate the cells counted. The resulting smear is stained on the Wescor stainer and evaluated microscopically. In addition, the color and transparency is recorded by visual inspection by the technologists. The CSF protein is measured on the Roche Hitachi P module.

### RFFIT

Blood and CSF samples were collected for measurement of VNA using the RFFIT (Rapid Fluorescent Focus Inhibition Test) as described previously [20]. Briefly, 50 µl of serial five-fold dilutions of serum were prepared in Lab-Tek Chamber slides (Nalge Nunc International, Rochester, NY). Fifty FFD_50_ (50% Fluorescing Foci dose) of CVS-11 was added to each chamber and incubated for 90 min at 37°C. NA cells (10^5^ cells) were added into each chamber and the slides were incubated at 37°C for 20 hr, fixed with ice-cold 80% acetone and stained with FITC-conjugated anti-RABV N antibodies for 1 hr at 37°C. Twenty fields in each chamber were observed under a fluorescent microscope, and the 50% endpoint titers were calculated according to the Reed-Muench formula. The values were compared with that of reference serum (obtained from the National Institute for Biological Standards and Control, Herts, UK) and normalized to international units (IU/ml).

### Ethics statement

The project AUP is entitled, “Virus clearance from the central nervous system” and the AUP number is A2011 03-016. It was approved by the University of Georgia's Institutional Animal Care and Use Committee on 4 APR 2011, and will expire on 4 APR 2014. The University of Georgia's University Research Animal Resources unit is fully accredited by the Association for Assessment and Accreditation of Laboratory Animal Care, International (AAALAC-I). The registration number from the U.S. Department of Agriculture, Animal and Plant Health Inspection Service, Animal Care is (USDA APHIS-AC). We have an assurance on file with the NIH-Office of Laboratory Animal Welfare (NIH-OLAW), and are in compliance with the PHS Policy on Humane Care and Use of Laboratory Animals and the 8th edition of the Guide for the Care and Use of Laboratory Animals, 2011.

### Statistical analysis

Statistical significance of the differences between groups was tested using student's T test with *** indicating a p value <0.0001, ** a p value <0.001, and * a p value <0.05 using GraphPad prism software.

## Results

### Clinical observations of dogs after infection with DRV-Mexico

Among the eight dogs infected with DRV-Mexico, three dogs showed typical signs of the dumb or paralytic form of rabies at 11, 12, and 21 days post infection (dpi), respectively. Early signs consisted of subtle changes in behavior, including quietness, hiding in the corner of the cage, lethargy and loss of appetite. Later signs included agitation, poor coordination, tremors, trembling, persistent regurgitation and retching, excess salivation and paralysis. Among these dogs, only one showed signs of furious form of rabies such as aggressiveness, whining and barking. The rabid dogs reached the end point of the study (hind limb paralysis) and were sacrificed on 13, 14, and 22 dpi, respectively. The other 5 infected dogs exhibited only the subtle behavior changes, which began on 13 dpi, and appeared normal again by 21 dpi. At 30 dpi, all surviving dogs were sedated and after blood and CSF collection were euthanized and their brains removed for analysis. None of the dogs in the vaccinated group (n-4) showed any clinical signs indicative of rabies infection during the 30 day observation period after which they were euthanized.

### VNA responses in the serum and the CSF in the dogs

**Figure 1:**
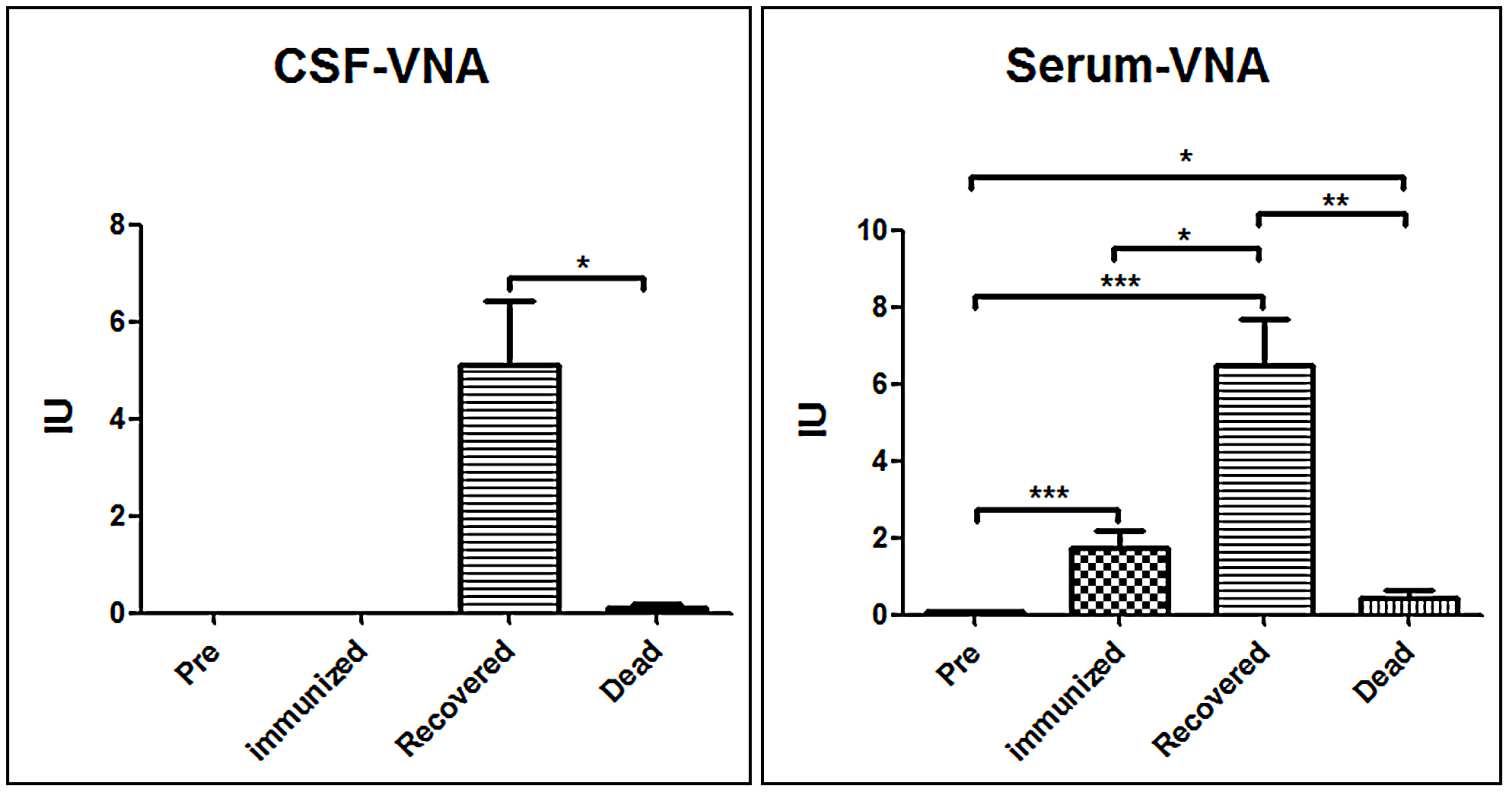
Detection of VNA in the serum and CSF. CSF and serum samples were subjected to RFFIT for the analysis of virus neutralizing antibodies (VNA). Statistical analysis was performed by student T test and the symbol *** indicates a p value <0.0001, ** indicates a p value <0.001 and * indicates a p value <0.05.

All the serum and CSF samples were subjected to RFFIT analysis for VNA. None of the dogs had detectable VNA in either the serum or the CSF prior to infection or vaccination. Serum and CSF VNA titers detected in terminally ill dogs at the time of euthanization were only 0.42±0.41 and 0.1±0.15 IU/ml, respectively. VNA titers determined in the serum and CSF of the surviving dogs were 6.5±2.6 and 5.1±2.9 IU, respectively, at 30 dpi (Fig. 1 & table 1). The vaccinated group had serum VNA titers of 21.5±9.7 IU prior to challenge with 1.75±0.9 IU in serum and no detectable VNA in CSF at 30 dpi (Fig. 1 & table 1). The high VNA levels in the serum of the dogs recovering from clinical signs of rabies indicates that they were infected with the virus and the presence of VNA in CSF strongly suggests that the virus invaded their CNS, and VNA contributed to their survival. Dogs that developed lethal rabies did not develop detectable VNA levels in CSF.

**Table 1:** Summary of immune responses in dogs.

Dog ID	CSF-WBC/µl		CSF-Total Protein-mg/dl		Serum VNA –IU			CSF VNA –IU		Status
	Pre	End of study	Pre	End of study	Pre	Immunized before challenge	End of study	Pre	End of study	
CEM	5	256	13.6	72.8	0.03	-	0.85	0	0.29	Dead
CLJ	0	363	10.1	66	0.07	-	0.35	0	0.02	Dead
CIJ	2	408	12.6	54.4	0.07	-	0.05	0	0.01	Dead
CFA	2	23	11.8	26.8	0.1	-	9.85	0	7.79	Recovered
CFB	0	33	12.2	32	0.2	-	7.5	0	2.6	Recovered
CGW	2	39	13.7	42	0.06	-	7.5	0	7.79	Recovered
CGT	1	21	13.9	19.7	0.09	-	3.3	0	1.5	Recovered
CGU	3	29	13.9	27	0.06	-	4.35	0	5.92	Recovered
CEH	ND	6	ND	14.5	0.01	9.85	1.7	ND	0	vaccinated
CEN	ND	8	ND	13.4	0.01	29.6	1.7	ND	0	vaccinated
CEZ	ND	8	ND	13.7	0.01	29.6	2.9	ND	0	vaccinated
CII	ND	0	ND	13.1	0.02	17.1	0.7	ND	0	vaccinated

Summary of total protein, virus neutralizing antibodies and/or WBC counts in the CSF and serum, of rabid, recovered and vaccinated dogs. ND: Not done.

### Total protein level and WBC counts in the CSF of dogs

**Figure 2:**
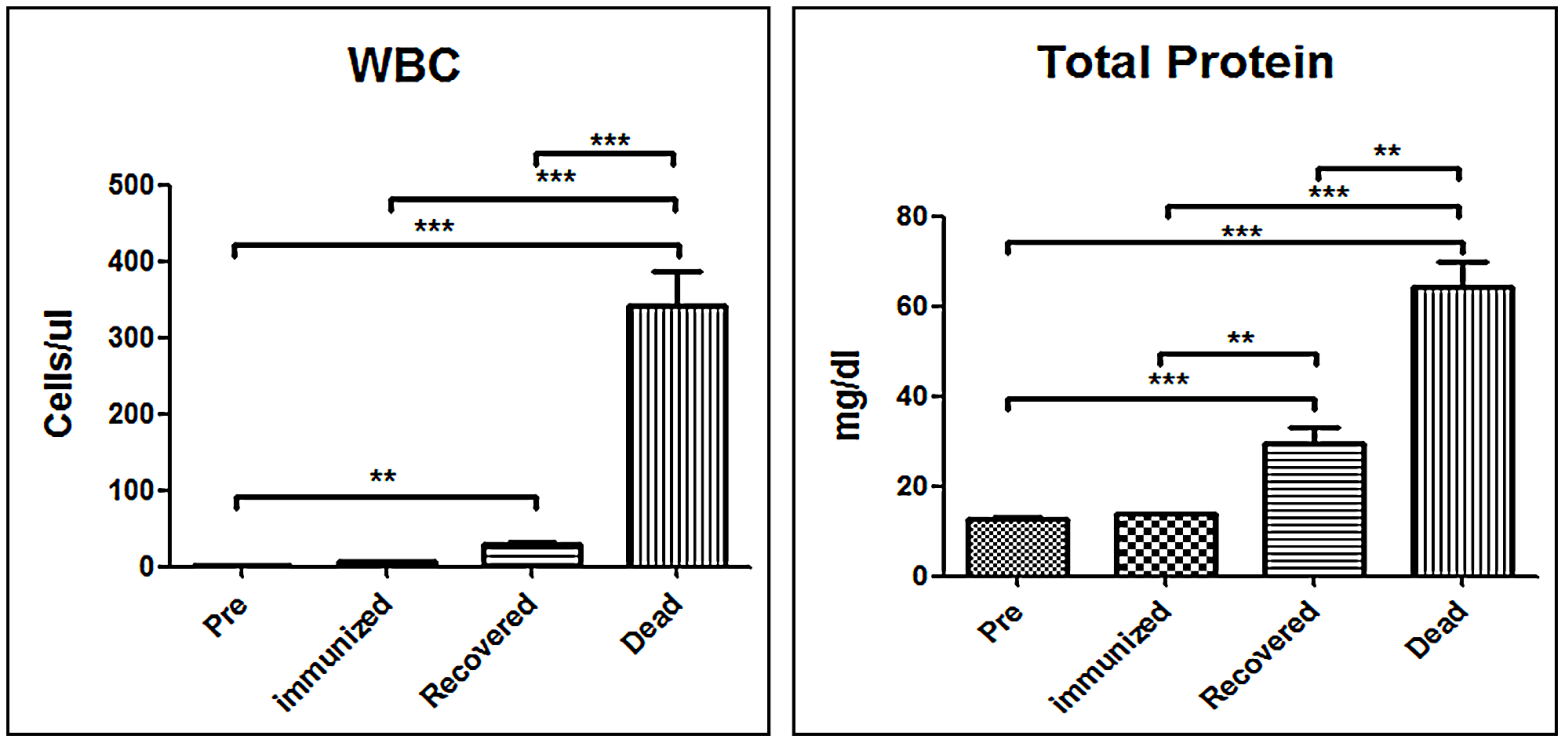
Measurement of WBC and total protein in the CSF. CSF samples obtained from DRV Mexico infected dogs were subjected to clinical pathological analysis. Statistical analysis was performed by student T test and the symbol *** indicates a p value <0.0001, ** indicates a p value <0.001 and * indicates a p value <0.05.

Total protein and WBC counts in the CSF were also analyzed. As shown in Fig. 2, no WBC were detected in the CSF and total protein was 12.7±1.3 mg/dl in dogs prior to infection or in the immunized dogs, indicating that the BBB remained intact in these animals. Total proteins (64.4±9.3 mg/dl) and WBC counts (342.3±78.1 cells/µl) increased dramatically in the CSF of terminally ill dogs, indicating a strong CNS inflammatory response (Fig. 2 & table 1). In the surviving dogs, total proteins (29.5±8.2 mg/dl) and WBC counts (29.0±7.3 cells/µl) in CSF were higher than those in the dogs prior to infection or in the immunized animals, but were much lower than those in the terminally ill animals. The relatively large amount of WBC and proteins in CSF suggest that BBB permeability is enhanced in DRV-Mexico-infected dogs.

### Pathology and infiltration of inflammatory cells in the CNS of dogs

**Figure 3:**
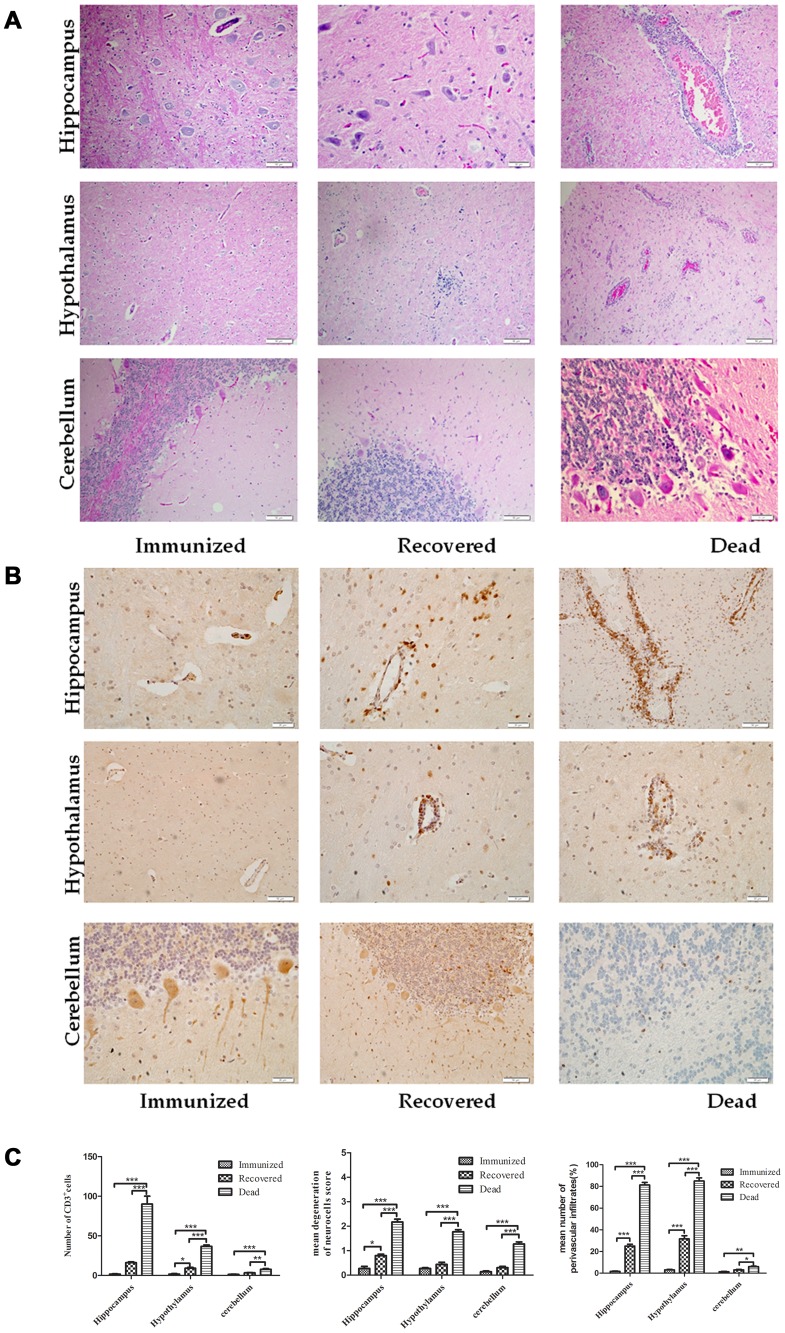
Infiltration of inflammatory cells into the CNS of dogs. Paraffin sections obtained from DRV Mexico infected dog brains were subjected to HE staining (A) or immunohistochemistry for detecting CD3-positive cells (B). Three serial sections and two vessels were selected from each dog for quantification, and the average numbers of CD3-positive cells, degenerated neurons and perivascular infiltrated cells obtained were used for statistical analysis (C). Statistical analysis was performed by student T test and the symbol *** indicates a p value <0.0001, ** indicates a p value <0.001 and * indicates a p value <0.05.

To determine whether the apparent changes in BBB permeability were reflected in the infiltration of immune/inflammatory cells into the brain, brains were collected from the immunized, surviving and terminally ill animals for histopathology and immunohistochemistry. Increased cell infiltrates and perivascular cuffing of mononuclear cells with marked activation of microglial cells were found in the hippocampus, hypothalamus and cerebellum of dogs that succumbed to rabies. Only residual cell accumulation was observed in the recovered dogs while evidence of inflammation was absent in the immunized dogs (Fig. 3A). To quantify immune cell infiltration into the CNS, CD3-bearing cells were assessed in various brain regions by immunohistochemistry. As shown in Fig. 3B, more CD3-positive cells were found in the hippocampus and hypothalamus of dogs that succumbed to rabies than in the same regions of recovered dogs. CD3 positive cells were not observed in the CNS tissues of immunized dogs (Fig. 3B). Quantification of CD3-positive cells in different parts of the brains of the groups of animals indicates that there were indeed significantly more CD3+ cells in the CNS of the dogs that succumbed to rabies than in those that survived (Fig. 3C).

### Viral antigens in the CNS of dogs

**Figure 4:**
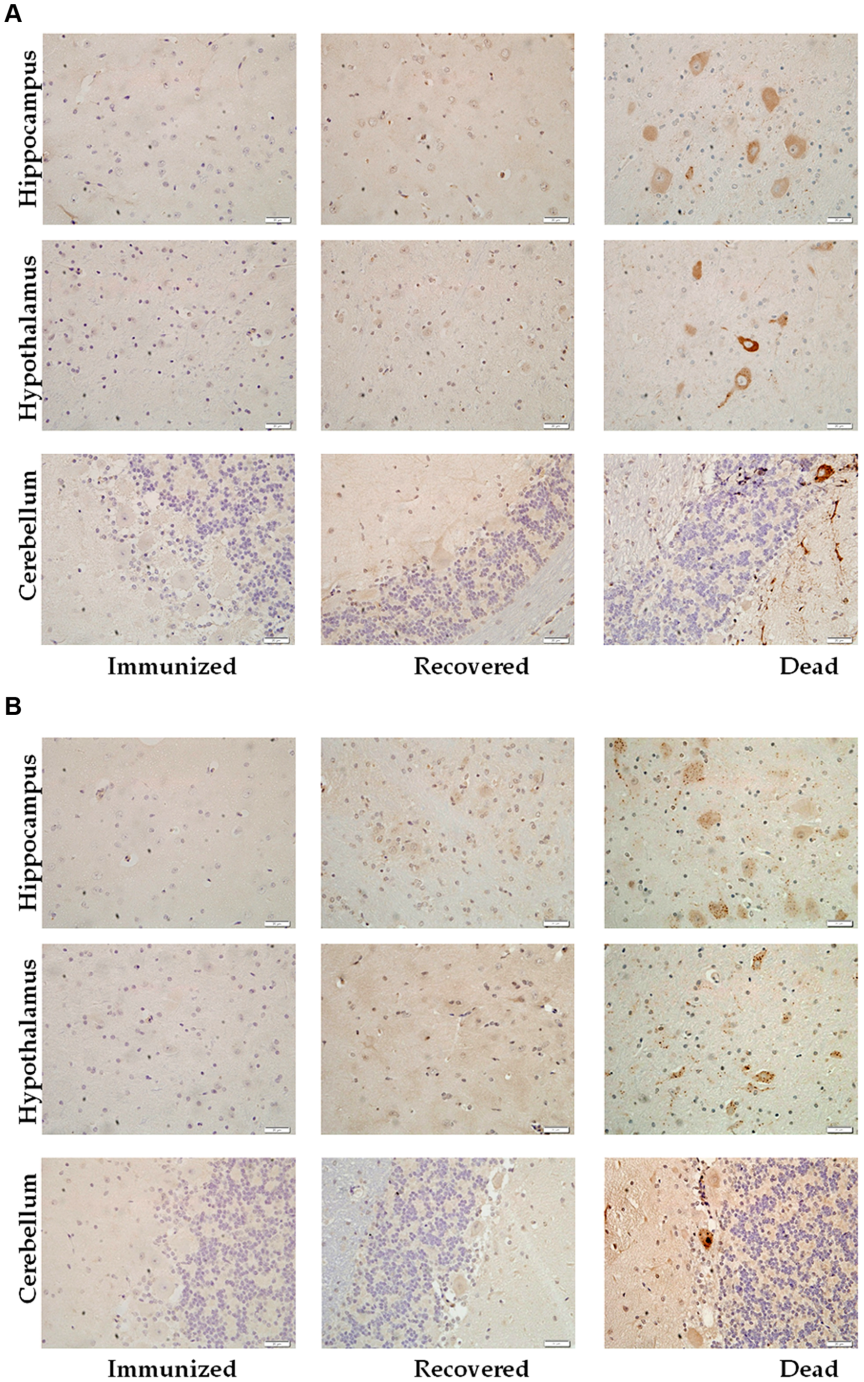
Detection of viral antigens in dog brain. Paraffin sections obtained from DRV Mexico infected dog brains were subjected to immunohistochemistry for the detection of RABV glycoprotein (A) and nucleoprotein (B) in the hippocampus, hypothalamus and cerebellum.

In order to correlate the development of rabies with virus loads in the CNS, brain tissues were evaluated for viral G and N antigens by immunohistochemistry. As shown in Fig. 4, viral antigens were detected only the hippocampus, hypothalamus and cerebellum of the dogs that succumbed to rabies. Relatively high numbers of cells positive for rabies G (Fig. 4A) and N (Fig. 4B) were detected in the hippocampus and hypothalamus, whereas such were only sparsely detected in the cerebellum. No viral antigens were detected in the CNS of recovered or immunized dogs.

## Discussion

Despite the extensive progress in rabies research since the time of Pasteur, more than 55,000 people continue to die of rabies each year world-wide [28]. Once symptoms occur, rabies is almost always fatal. Recently, however, cases of non-lethal rabies infection and recovery from clinical rabies in laboratory animals and humans have been reported [6], [10], [21], [29]–[32]. One of the major findings associated with these non-lethal infections is that many of the survivors had VNA in the CSF [6], [10], [22], [29], [32], [33]. Consistent with these findings, we report in the present paper that experimental infection of dogs with a wt RABV is not invariably lethal, and that survival correlates with the presence of high VNA titers, evidence of WBC infiltration, and elevated levels of protein in the CSF.

PEP is effective in preventing rabies after exposure providing that clinical signs of rabies have not appeared. Consisting of vaccination with RABV vaccines and administration of anti-rabies immunoglobulin at the site of exposure and systemically [34], it is believed that PEP prevents the rabies virus from invading the CNS due to the long incubation period of the infection [35]. It has long been thought that it is difficult to clear the virus once it enters into the CNS [4], [36]. This dogma was initially cast into doubt by the finding that RABV can be cleared form the CNS by VNA administered intravenously [37]. Recently it has been found that enhancement of the BBB permeability prevents rabies in the mouse model by allowing immune effectors from the periphery to enter into and clear RABV from the CNS [16]. Various studies have been conducted using different models to promote immune cell infiltration into the CNS in rabies including experimental allergy encephalitis, in which the disease enhances BBB permeability [38], and infection with attenuated RABV such as recombinant viruses that express three copies of G [39] or immune stimulating agents [40].All of these interventions result in an immune response to RABV in the CNS and reduce fatalities in mice infected with wt RABV [17]–[19].

However, simply targeting BBB integrity alone is not sufficient to protect mice from lethal rabies since administration of a chemokine, MCP-1, enhanced BBB permeability but did not significantly increase the survival rate of mice infected with DRV-Mexico [41]. Neither does immunization with an inactivated RABV preparation 5 days after infection with DRV-Mexico despite inducing serum VNA. On the other hand, administration of MCP-1 to mice immunized with inactivated RABV significantly improved their survival from DRV-Mexico indicating that the combined effects of enhancement of BBB permeability and the production of rabies-specific VNA is protective [41]. In the present study, none of the dogs that succumbed to rabies had serum VNA = />0.5 IU and their CSF VNA were even lower (∼0.1 IU). This is despite the possibility that BBB permeability became enhanced at the end of their lives as suggested by the greater numbers of WBC and high protein levels detected in their CSF. In the dogs with non-lethal infection, BBB permeability had likely been enhanced at some stage since VNA, WBC, and high protein levels were detected in their CSF. Moreover, residual CD3 cell accumulation was observed but no virus antigen in the CNS tissues of these dogs at the time of sacrifice, suggesting that immune effectors acting in the CNS had cleared the virus. In the mouse model, lethal infection with wt RABV (DRV-Mexico) is not accompanied by enhanced BBB permeability (cerebrum, cerebellum, or spinal cord) at days 6 or 9 dpi when RABV antigens or RNA are detected in the CNS [19]. This is consistent with observations in mice of lethal infections with a variety of other wild-type RABV [42]. The apparent discrepancies observed between mice and dogs in the loss of BBB integrity indicate that the pathogenic processes in these two animal species may be somewhat different. Also, these discrepancies in BBB permeability may be due to the close co-adaptation of viral strain to their specific homologous host (dogs) than the spillover. Nevertheless, the correlation between the presence of VNA in the CSF and the clearance of RABV from the CNS is shared. In 3 of the humans that recovered from rabies, whether treated with the Milwaukee protocol or not, virus specific antibodies were detected in their CSF at the time of hospitalization [10], [29], [43]. In dogs recovered from infection with laboratory-attenuated virus, VNA was also detected in the CSF [44]. A ferret that recovered from rabies encephalitis also had detectable VNA in the CSF [32]. In our study, the dogs surviving wt RABV infection developed only mild disease without typical rabies symptoms yet developed high VNA levels in both serum and CSF. Particularly, the presence of VNA in CSF strongly suggests that the virus invaded their CNS. This is very different from immunized dogs in which VNA was produced in the serum, but not in the CSF. The detection of VNA in CSF and evidence of limited immune cell infiltration into CNS tissues makes it likely that the dogs survived the wt RABV infection by clearing the virus from the CNS. Thus we conclude that, like humans and other species [8], [9]–[14], [43], infection of dogs with wt RABV is not invariably lethal even when the virus has reached the CNS. The mechanisms whereby certain dogs mediated an RABV-specific immune response that reached in the CNS and survived while others did not remain to be understood and may provide the foundations for the development of novel therapeutic intervention strategies for clinical rabies.
